# An efficient depth map preprocessing method based on structure-aided domain transform smoothing for 3D view generation

**DOI:** 10.1371/journal.pone.0175910

**Published:** 2017-04-13

**Authors:** Wei Liu, Liyan Ma, Bo Qiu, Mingyue Cui, Jianwei Ding

**Affiliations:** 1 Electromechanic Engineering College, Nanyang Normal University, Nanyang, Henan, China; 2 Center for Internet of Things, Institute of Microelectronics of Chinese Academy of Sciences, Beijing, China; 3 Electronic and Information Engineering College, Hebei University of Technology, Tianjin, China; 4 Information Technology and Network Security College, People’s Public Security University of China, Beijing, China; Beijing University of Technology, CHINA

## Abstract

Depth image-based rendering (DIBR), which is used to render virtual views with a color image and the corresponding depth map, is one of the key techniques in the 2D to 3D conversion process. Due to the absence of knowledge about the 3D structure of a scene and its corresponding texture, DIBR in the 2D to 3D conversion process, inevitably leads to holes in the resulting 3D image as a result of newly-exposed areas. In this paper, we proposed a structure-aided depth map preprocessing framework in the transformed domain, which is inspired by recently proposed domain transform for its low complexity and high efficiency. Firstly, our framework integrates hybrid constraints including scene structure, edge consistency and visual saliency information in the transformed domain to improve the performance of depth map preprocess in an implicit way. Then, adaptive smooth localization is cooperated and realized in the proposed framework to further reduce over-smoothness and enhance optimization in the non-hole regions. Different from the other similar methods, the proposed method can simultaneously achieve the effects of hole filling, edge correction and local smoothing for typical depth maps in a united framework. Thanks to these advantages, it can yield visually satisfactory results with less computational complexity for high quality 2D to 3D conversion. Numerical experimental results demonstrate the excellent performances of the proposed method.

## Introduction

Nowadays three-dimensional (3D) films are becoming more and more popular due to their higher realism over the conventional two-dimensional (2D) ones. The prosperity of 3D industry has lead to a growing demand for 3D contents. The traditional approach of constructing stereoscopic images uses two or multiple cameras [1] to capture two or more streams of images and transmit them to receiver for viewing purposes. This scheme allows to transmit and to reproduce for the user the information that would have been received in the real life. In fact, the majority of material available for 3D TV broadcast today has been produced in this way. However, shot planning, costly hardware, camera rig control, and extensive post-processing required to fix stereographic errors render this process both cumbersome and expensive.

On the contrary, Depth Image Based Rendering (DIBR) techniques, which only use one color image and the corresponding per-pixel depth information to synthesize virtual view according to the requirement of various stereoscopic devices, is regarded as another promising solution by the European Information Society Technologies (IST) project “Advanced Three-Dimensional Television System Technologies” (ATTEST) [2]. The depth image is a 2D grey scale image in which each pixel indicates the corresponding depth in the intermediate image [3]. There are many ways to generate 3D contents. Depth map can be captured by active approaches with range devices such as Zcam, which uses the time of flight concept to measure the depth of a scene. In comparison with stereo and multiple camera systems, a depth camera can handle varying conditions more easily [4]. For the passive approaches, the depth camera is replaced by a 2D to 3D converter, where depth information is extracted from a monoscopic image sequence using computer vision technologies [5]. On one hand, this helps to reduce the overall cost of the system. More importantly, it enables the large existing libraries of 2D program material to be reused. As a result, this solution provides a practical way to solve the bottleneck of 3D contents for DIBR-enabled 3D media applications. When the depth map is available, DIBR systems can generate any number of views without multi-camera systems, thus the equipment cost of 3D cinema systems is reduced.

The image plus depth data format, which is adopted by the DIBR techniques, has several advantages compared to the conventional broadcasting system. The transmission bandwidth required by the image plus depth data format can be reduced at least 33% in comparison to that of two color images [6]. Another advantage is that the DIBR system allows users to customize the parallax of generated virtual view images to achieve different depth effects and experience different 3D perceptions. Also, being backward compatible with 2D TV, the DIBR system provides users display selectively between 2D or 3D display methods. Theoretically, utilizing each depth value of the object in the transmitted image, the DIBR techniques can be used to synthesize any virtual perspective views from the image plus depth data. However, due to sharp horizontal changes in the depth map, the image warping may reveal areas that are occluded in the original view and become visible in some virtual views. To deal with that problem and achieve high quality 3D, these holes should be filled.

Lots of algorithms have been developed to solve this problem. One solution would be to rely on more complex multi-dimensional data representations, like Layer Depth Image (LDI) data representation [7], which can achieve excellent rendering results by providing sufficient information of the scene. LDI data allows to store additional depth and color values for pixels that are occluded in the original view. This extra data provides the necessary information that is needed to fill in disoccluded areas in the rendered, novel views. It is very simple to obtain high quality multi-view images from LDI data. However, the procedure of creating LDI is computationally complex and quite time-consuming. Besides that, in the 2D to 3D conversion process, disocclusions removal can be achieved in pre-processing step by filtering the depth map in order to reduce depth data discontinuities such that holes are avoided in the first place rather filled later, or in post-processing step by image inpainting techniques to close holes after they have appeared in a texture image [8]. Specially, if video sequences are provided, temporal correlation between different frames of the intermediate view can be used to improve the synthesis. In a previous work [9], motion vectors were computed and used to retrieve information about disoccluded regions from other frames. In another work [10], background information was extracted with adjacent views from multiple time instants and used for hole filling in a DIBR synthesis.

Besides that, if the edges of objects in depth or disparity map are not accurate, algorithms based on image or video segmentation are always used to classify the edges of objects for a good depth or disparity map. However these strategies cost a lot of time. Although method [11] can correct the edges when smoothing a depth map, a reference fame in the video must be provided.

To better deal with the problems of disocclusion occurrences, edge inconsistency and over-smoothness in the DIBR process, this paper proposes an efficient structure-aided depth map preprocessing method. Compared to other similar previous works, the main novel advantages of our work are:

- The presented framework is based on domain transform, which uses a dimensionality-reduction strategy and has some desirable features, such as considerable speed-ups over existing techniques and computational cost not affected by the choice of filter parameters. Therefore, the proposed filtering scheme in the transformed domain is not only more efficient, but also more optimal than other similar works when hybrid constraints are considered.
- Advanced than other similar works, our framework realizes hole filling using both structure and visual saliency information to smooth out deformations. By this way, the smoothing strategy can be adjusted adaptively according to the scene structures around, and the artifacts in salient image regions can be reduced. Besides that, edge correction and local smoothing of the depth maps are also achieved in this united framework simultaneously. Thanks to these advantages, our method makes good performance both on computational cost and virtual view quality, and is more suitable for the 2D to 3D conversion application.

The rest of the paper is organized as follows. Firstly, previous works and theoretical backgrounds about DIBR and hole filling in this process will be presented. Then, the framework of the proposed method will be introduced. The features of our structure-aided filter in the transformed domain, including structure-aided smoothness with depth correction, visual saliency weighted hole filling and adaptive smooth localization, will be discussed respectively and elaborately. Finally, experimental results and discussions will be reported, and some concluding remarks will be given. Experiments and comparisons show that our methods are both excellent at time saving and virtual view quality.

## Previous works and theoretical backgrounds

### Depth image-based rendering

**Fig 1:**
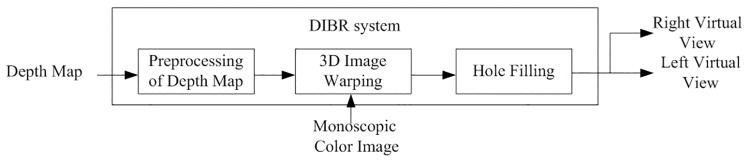
Block diagram of the DIBR system.

A DIBR system usually includes preprocessing of depth, image warping and hole filling as illustrated in Fig 1. The depth map preprocessing step processes the depth value in a depth map to reduce hole occurrences, and hole filling step is designed to deal with the newly exposed areas known as holes (or disocclusions) after 3D image warping. Technical details about these two steps will be discussed in the next sub-section.

The core process of synthesizing virtual views of a real-world scene based on DIBR is commonly referred to as 3D warping [12], which can be understood as a two-step procedure: first, the original view points are re-projected into 3D world using depth values (2D to 3D), then these intermediate 3D space points are projected into the virtual image plane (3D to 2D).

**Fig 2:**
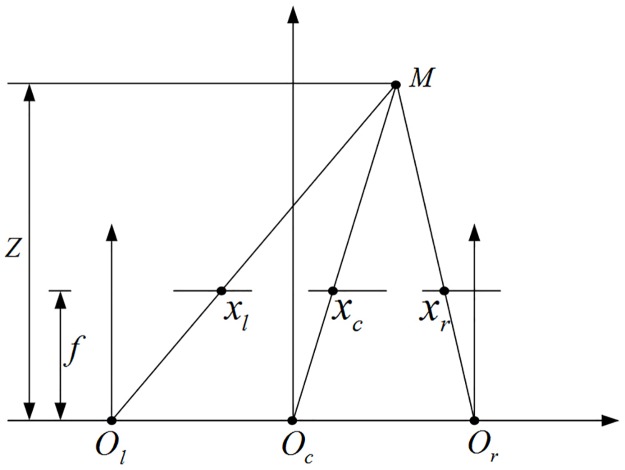
Parallel camera configuration used for the generation of virtual stereoscopic images.

Real stereo cameras typically use two different configurations, the *toed-in* approach and the *shift-sensor* approach (or parallel configuration). The parallel configuration is preferred to give a better viewing experience because it does not produce vertical difference which is the cause of eye-strain [13]. As shown in Fig 2, the transformation that defines the new horizontal coordinate in the left view *x*_*l*_ and right view *x*_*r*_ from the reference image at *x*_*c*_ according to the 3D space point *M* is calculated as:

$$\begin{array}{c} x_{l}=x_{c}+\frac{t_{x}}{2}\frac{f}{Z}\\ \begin{array}{c} x_{r}=x_{c}-\frac{t_{x}}{2}\frac{f}{Z} \end{array} \end{array}$$ (1)

where the horizontal camera translation *t*_*x*_ here is equal to the average human eye separation, *f* is the focal length of the reference camera, and *Z* is the depth distance from the 3D space point *M* to the left (*O*_*l*_) or right (*O*_*r*_) cameras. The formula shows that in the parallel configuration, pixels of intermediate view to left and right view are only mapped in horizontal direction.

The disparity *d*, which is defined as the distance between the horizontal coordinates, is found inversely proportional to the depth $Z:d=\frac{ft_{x}}{Z}$. So Eq (1) can also be expressed as:

$$\begin{array}{c} x_{l}=x_{c}+\frac{d}{2}\\ \begin{array}{c} x_{r}=x_{c}-\frac{d}{2} \end{array} \end{array}$$ (2)

It should be mentioned that the disparity used in stereo conversion is measured in pixels. The transform formula between pixel level and actual distance in real scene can be found in [14], which depends on multiple viewing conditions. Because a disparity value and the corresponding depth value have inversely proportional relationship as shown above, for notational convenience, we do not distinguish disparity and depth in this paper any more. For the remainder of this paper, the depth map is represented as an 8-bits gray-scale image. The continuous depth range is quantized to 255 discrete depth values. The nearest object to the camera image sensor is assigned with 255 and the farthest object is assigned with 0.

### Hole filling in DIBR process

In this paper, we mainly focus on stereoscopic view synthesis in the application of conventional 2D to 3D conversion process. As general 2D to 3D conversion cannot rely on multiple camera depth estimation or a priori known depth maps, depth has to be estimated from a single view. No more complex multi-dimensional data like the LDI format can be used, so it is more difficult to deal with the disocclusion problem. It should be noted that this paper assumes that only an original texture image and its corresponding depth map, which has been estimated with some depth cues, are provided. Research of stereoscopic view synthesis for video sequences would be carried out in our further work. Here we do not discuss stereoscopic video synthesis with temporal prediction like works [9, 10] deeply.

Generally speaking, the existing DIBR methods which can cope with disocclusions always follow the procedure flow as we have discussed in Fig 1. According to the different steps these approaches emphasize when realizing hole filling in the DIBR process of 2D to 3D conversion, we categorize those methods into two streams. The first one focuses on virtual image inpainting after DIBR. These methods always do more works to achieve hole-filling utilizing auxiliary information around predicted hole regions by either texture replication or structure continuation after DIBR. Since an in-depth discussion of inpainting is beyond the scope of this paper, interested readers are referred to a summary of these methods [15]. However, inpainting is a challenging problem, and it is more difficult with stereoscopic content as image features need to have consistent disparity across the two generated views. The second one focuses on preprocessing of depth maps before DIBR. These methods always emphasize to use depth map smoothing before DIBR to avoid holes. Previous research results [16] demonstrate that smoothing of depth maps is beneficial to reduce the percentage of disoccluded areas which are required to be filled in the rendering process. To remove sharp discontinuities from depth image, Tam et al. [17] adopt symmetric Gaussian filter to smooth depth maps. Therefore, the artifacts on boundaries of objects are reduced after warping process. However, symmetric filter may produce distortions in some areas depending on the depth values of neighboring regions, especially for that around vertically straight object boundaries. To solve this problem, several methods are proposed, such as asymmetric smoothing algorithm [18] and some edge-oriented filters [19, 20]. The asymmetric smoothing algorithm aims to keep stronger smoothing in vertical direction, while the edge-oriented filters improve both the depth quality and the virtual view quality through minimization of filtered regions. However, geometric distortions in some severe sharp depth discontinuities of the filtered areas are still inevitable.

In this paper, a new method for the preprocessing of depth maps to prevent holes during the view synthesis is proposed. Our method is inspired by the work in [8], where hybrid cues including visual saliency and structure information were also adopted to exploit the depth map smoothness underlying optimization problem. Advanced than the previous works, the method presented in this paper builds a more efficient framework based on domain transform for using even more constraints to simultaneously deal with the problems of disocclusion occurrences, edge inconsistency and over-smoothness in the DIBR process, while other methods always need more separate steps to realize.

### Domain transform

Domain transform [21] is a simple transform that can preserve the geodesic distances between points on the processed image curves, and adaptively warp the input signal so that 1D edge-preserving filtering can be efficiently performed in linear time. When applying domain transform, one image can be filtered in the transformed domain by iterating 1D-filtering operations only in row or column order rather than filtering it in a two dimensional manner, which is the main cause of heavy computational burden. Consequently, with low computational complexity and distance preserving property, domain transform is extremely efficient for processing some content-aware real-time image and video processing tasks, such as recoloring, colorization, tone mapping, etc. In this paper, we try to build a new framework using this technique in the application of conventional 2D to 3D conversion process.

The multichannel transformation can be expressed as:

$$ct\left(u\right)=\int_{0}^{u}1+\sum_{k=1}^{c}\left|{I^{\prime }}_{k}\left(x\right)\right|dx$$ (3)

where *I*_*k*_(*x*) is the *k*-th channel of the signal. When taking the signal’s space and range into considerations, the domain transform can be further expressed as:

$$ct\left(u\right)=\int_{0}^{u}\frac{\sigma_{H}}{\sigma_{s}}+\sum_{k=1}^{c}\frac{\sigma_{H}}{\sigma_{r_{k}}}\left|{I^{\prime }}_{k}\left(x\right)\right|dx$$ (4)

where *σ*_*H*_, *σ*_*s*_, *σ*_*r*_*k*__(*k* = 1, …*c*) are parameters to control the smooth effect of the result. After performing domain transform, we should design a filter in the transformed domain to carry out image filtering. In our proposed framework, hybrid constraints are regarded as special multiple channels of the signal, and which will be discussed elaborately in the next section.

## The proposed method

### Framework of the proposed method

The core idea of the original domain transform filter is similar to bilateral filter, which is a non-linear technique that can blur an image while preserving strong edges. In this paper, as discussed in the above sub-section, to incorporate more related cues into the process of 2D to 3D conversion, we view a pixel of an image in a high dimensional space, which is formed by coordinate, pixel texture, depth, visual saliency and influence information. After we extend the original domain transform into this new framework, we can express it with a recursive form in the transformed domain as:

$$J\left[n\right]=\left(1-a^{d}\right)I\left[n\right]+a^{d}J\left[n-1\right]$$ (5)

where *I*[*n*] is a pixel value in a row or column of the original depth map. *d* = *ct*(*x*_*n*_) − *ct*(*x*_*n* − 1_) is the distance between neighbor samples *x*_*n*_ and *x*_*n* − 1_ in the transformed domain. The feedback coefficient of this filter is computed as $a=e^{\frac{-\sqrt{2}}{\sigma_{H}}}$ [21], where *σ*_*H*_ is the standard deviation of the desired filter. Since *a* ∈ [0, 1], the filter is stable, and has a complexity of *O*(*N*).

In Eq (5), *d* controls smooth strength delivered from transformed domain to depth maps. When *d* increases, *a*^*d*^ goes to zero, stopping the propagation chain. Thus, smoothing effect adjusts adaptively according to the related constraints that we have defined in the transformed domain. The smooth process in Eq (5) firstly performs a horizontal pass along each image row, and then a vertical pass along each image column. We notice that the impulse response of Eq (5) is not symmetric. To achieve symmetric ones, Eq (5) is performed left-to-right (top-to-bottom) in one iteration, then in the next iteration right-to-left (bottom-to-top). The required number of horizontal and vertical passes depends on the image content. In this paper, the process is performed four iterations for each depth map.

**Fig 3:**
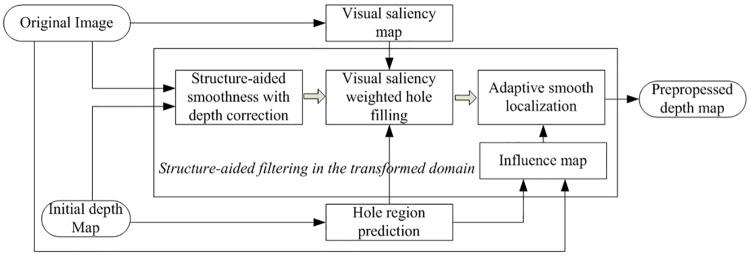
Framework of the proposed structure-aided depth map smoothing approach.

Fig 3 shows the framework of proposed structure-aided depth map smoothing approach. We can observe that our filter incorporates three different constraints in the transformed domain, which are:

- *ct*_1_(*u*): Structure-aided smoothness with depth correction
- *ct*_2_(*u*): Visual saliency weighted hole filling
- *ct*_3_(*u*): Adaptive smooth localization

For notion convenience, the filter in the transformed domain is further expressed in the following form. And then, we will discuss the details elaborately in the next sub-section.

$$ct\left(u\right)=ct_{1}\left(u\right)+ct_{2}\left(u\right)+ct_{3}\left(u\right)$$ (6)

### Structure-aided filtering in the transformed domain

#### Structure-aided smoothness with depth correction

The first part of the proposed filter in the transformed domain mainly realizes the structure-aided smoothness. As shown in Eq (7), this part can be further described by two terms. *ct*_*I*_(*u*) is the texture term, which is actually an simplified version of the original domain transform as we have discussed in Eq (4). *ct*_*c*_(*u*) is an additional term, which is used to extend the framework for realizing depth correction when smoothing depth maps. In the experiments of this paper, the generated 3D views are synthesized anaglyph images for each test set. For this reason, the corresponding texture image *I*(*p*) here is only one channel of the original color image, and is also used as the original view image to be processed. Previous research results [18] have demonstrated that asymmetric smoothing is an efficient way to reduce geometric distortions. To achieve *asymmetric* in our domain transform filter, the size of 3 × *σ*_*s*_ is used to enhance smooth effect in space when performing a vertical pass along each image column.

$$ct_{1}\left(u\right)=ct_{I}\left(u\right)+ct_{c}\left(u\right)$$ (7)

$$ct_{I}\left(u\right)=\int_{0}^{u}\frac{\sigma_{H}}{\sigma_{s}}+\frac{\sigma_{H}}{\sigma_{r}}\left|I^{\prime }\left(x\right)\right|dx$$ (8)

$$ct_{c}\left(u\right)=\int_{0}^{u}\sigma_{c}e^{-\alpha I_{c}\left(x\right)}dx$$ (9)

$$\begin{array}{c} I_{c}\left(p\right)=\left\{\begin{array}{c} \varepsilon_{1},\;if\;p\in G_{D}\;and\;p\in G_{I}\\ 1,\;\;\;\;otherwise \end{array}\right. \end{array}$$ (10)

To create a stereoscopic pair from the monoscopic input, it requires some notion of scene depth to generate a virtual view which is close to the original. Ideally, if accurate depth is available, such view synthesis can be implemented by approaches discussed in Fig 1. However, general 2D to 3D conversion cannot rely on multiple camera depth estimation or a priori depth maps, and depth has to be estimated from a single view. Although many approaches have been proposed using the information contained inside of an image, so-called *depth cues*, to try and determine pixel depth, it is still hard to reconstruct accurate edges of objects in depth maps, especially for the automatic approaches with certain single cues [5].

Our depth correction constraint can deal with this problem. In Eq (9), *σ*_*c*_ and *α* are positive parameters, and whose values can be adjusted when the system is operating. *I*_*c*_(*p*) in Eq (10) describes the consistency between a texture image and its related depth map, where *G*_*D*_ and *G*_*I*_ stand for the detected edges of them respectively. *ε*_1_ is a preset value, and we set *ε*_1_ = 0.2 in this paper. The main feature of depth image is that it contains abundant edge information of its related texture image. If a depth map is accurate, a pixel *p* on one object edge of it should be also located on one object edge of the related texture image. In this case, *I*_*c*_(*p*) got a small value *ε*_1_, which would further cause *ct*_*c*_(*u*) in Eq (9) and *ct*_1_(*u*) in Eq (7) to increase, and finally make the filtering strength in Eq (5) decrease. As a result, the original depth values are preserved in the last iteration. Contrarily, it should be smoothed to eliminate the fake edge. In this way, the term *ct*_*c*_(*u*) achieves depth correction when smoothing a depth map.

#### Visual saliency weighted hole filling

Generally speaking, the newly exposed holes would only appear after the process of DIBR. Here for the purpose of efficient hole filling in the depth preprocessed smoothing, the prediction of hole regions *R*_*H*_ is made previously. Firstly, we get the normalized disparity map from the depth map. A disparity map with sharp disparity discontinuities will lead to new holes occurring after image warping. For a left view, holes arise on the disparity map with small to big sharp disparity value transition and vice versa for a right view. Based on the above concept, *R*_*H*_ is predicted as:

$$\begin{array}{c} R_{H}=\left\{r\left(x,y\right)|\eta_{i}\left(x,y\right)-r\left(x,y\right)>k\cdot \frac{\lambda_{H}\cdot D_{max}}{D_{width}}\right\}\\ \eta_{i}\left(x,y\right)=\left\{\begin{array}{c} r(x+1,y)\;\text{if}i=l\\ r(x-1,y)\;\text{if}i=r \end{array}\right. \end{array}$$ (11)

where *r*(*x*, *y*) denotes the pixel in the disparity map at the location (*x*, *y*), *D*_*max*_ is the maximum horizontal distance of warped pixels in the virtual image, *k* is a normalization factor (e.g. *k* = 255 for a typical grey-level image). *D*_*width*_, measured in pixels, is the image width. *λ*_*H*_ is a preset weighting threshold which is set to be 2 in this paper. If the current synthesized virtual view is a left view, then *i* = *l*, otherwise *i* = *r*.

The second part of the proposed domain transform filter focuses on adaptive smoothing to fill the predicted holes. For this purpose, a visual saliency map is employed to indicate the importance of pixels. Basically, the underlying idea is to hide the image deformation in those regions which are not very important in an image, while leaving important (salient) regions intact. Therefore, we incorporate the visual saliency information into the filter to smooth out deformations. Based on the predicted holes in Eq (11) and normalized saliency map *s*(*p*) with the existing state of the art method from [22], *ct*_2_(*u*) can be expressed as:

$$\begin{array}{c} ct_{2}\left(u\right)=\int_{0}^{u}\sigma_{h}e^{-I_{H}\left(x\right)}+\beta \left|S^{\prime }\left(x\right)\right|dx\\ I_{H}\left(p\right)=\left\{\begin{array}{c} 1\;\text{if }p\in R_{H}\\ \varepsilon_{2}\;\text{if }p\notin R_{H} \end{array}\right. \end{array}$$ (12)

where *σ*_*h*_ is a positive parameter to control the effect of smooth, and *β* is a user controllable weight to determine the effect of visual saliency information to hole filling. *ε*_2_ is a preset value. In this paper, it is set to be 0.5. Similar to the analysis of *ε*_1_ discussed above, only in the case that pixels in the non predicted hole regions also have high visual saliency values, both *ct*_2_(*u*) and *ct*(*u*) in Eq (6) of the transformed domain will increase to further limit the smooth strength in Eq (5) and preserve the structure information in the original image. Contrarily, there will be corresponding smooth effect enhancement adaptively. In this way, we incorporate the visual saliency information into the filter to penalize local deformations, hiding artifacts in less salient image regions.

#### Adaptive smooth localization

If smooth is performed only in a depth map with sharp depth discontinuities around holes, computation time will be reduced and depth quality of non-hole regions will be preserved. This strategy has been used by edge-oriented methods [19, 20] successfully to reduce computational complexity when smoothing depth maps. However, widths of the smoothing regions are fixed when filters worked along the selected object edges around holes in those methods. This downside may still lead to geometric distortions in some severely sharp depth discontinuities of the filtered areas.

To further alleviate this problem, we define an influence map for each depth map to realize adaptive smooth localization. The final influence map is generated by blurring an initial influence map, which is defined as:

$$\begin{array}{c} I_{f-init}\left(p\right)=\left\{\begin{array}{c} 1-e^{-D_{e}\left(p\right)},\;if\;p\in R_{H}\\ 0,\;\;\;if\;p\notin R_{H} \end{array}\right. \end{array}$$ (13)

where *R*_*H*_ stands for hole regions which have been predicted by Eq (11), *D*_*e*_(*p*) is the distance from the point *p* to the edges of the predicted holes. Therefore, a large value indicates that the point is close to the center of predicted holes, while a small value indicates that point is far from the center. Obviously, it is believed that the central parts of the predicted holes need to be more emphasized when using preprocessed smoothing to fill holes. Taking *I*_*f* − *init*_(*p*) as the original image to be processed, the final influence map *I*_*f*_(*p*) is produced through joint filtering with the same approach operated by the recursive domain transform filter as discussed in Eqs (5) and (8). Then based on the final influence map, our method can realize adaptive smooth localization by adding the following constraint term to the proposed framework in the transformed domain.

$$ct_{3}\left(u\right)=\int_{0}^{u}\sigma_{l}e^{-\gamma I_{f}\left(x\right)}dx$$ (14)

Similar to the discussion on Eq (9), *σ*_*l*_ and *γ* are parameters which lead to enhance or eliminate the effect of smooth localization. If the influence map value in pixel *p* increases, smooth effect in Eq (5) is enhanced via the changes of the terms *ct*_3_(*u*) and *ct*(*u*) in Eq (6), vice versa. In this way, our method can adaptively limit the filtered regions according to the influence map in the united framework of the transformed domain, and it is outstanding than the edge-oriented methods.

## Experimental results and discussions

In this section, some experiments are carried out to further evaluate the performance of the proposed methods. Kinds of test sets, which cover a wide range of images with indoor and outdoor scenes, are used for evaluation including two publicly available ones with additional natural depth images (*Interview* [23] and *Ballet* [24]) and two ones with depth images generated by automatic 2D to 3D conversion methods (*Flower* [25] and *Castle* [26]). The experiments were implemented on a commodity PC with an Intel Core2 Quad CPUQ9400 2.66GHz. We implemented our proposed framework in Microsoft Visual Studio C++ 2008 platform combining this implementation with the domain transform module running in MATLAB. Experimental results are discussed in two sub-sections. The first sub-section is designed to show the experimental details at each of the core steps. In the second sub-section, our results are comprehensively compared with other state-of-the-art works and evaluated with qualitative and subjective criteria. It should be pointed out that all constants in the models of this paper are found in experiments. The parameter presets for the domain transform filter used in testing are given in Table 1. In these parameters, the presets of basic parameters such as *σ*_*s*_, *σ*_*H*_, *σ*_*r*_ are based on the analysises of previous works [21]. Since *σ*_*H*_ is a free parameter and the texture term in Eq (8) is only one constraints for the filter, we let *σ*_*s*_ = *σ*_*H*_ = 300. The presets of *ε*_1_ and *ε*_2_ have been discussed in the above section. For other parameters, *σ*_*c*_, *σ*_*h*_, *σ*_*l*_ are the weighting factors among different constraints, and *α*, *β*, *γ* can be used to further adjust the weighting fact slightly for each constraint. The parameter selection in Table 1, which can ensure the filter having good robustness for all test sets in our experiments, just provides an instruction.

**Table 1:** Parameter presets for the domain transform filter used in testing.

*ct*_1_(*u*)	*ct*_2_(*u*)	*ct*_3_(*u*)
*σ*_*s*_ = *σ*_*H*_ = 300	*σ*_*h*_ = 3	*σ*_*l*_ = 5
*σ*_*r*_ = 1, *σ*_*c*_ = 8	*β* = 2	*γ* = 1
*α* = 1	*ε*_2_ = 0.5	
*ε*_1_ = 0.2		

### Analysis of experimental results and depth map evaluation

**Fig 4:**
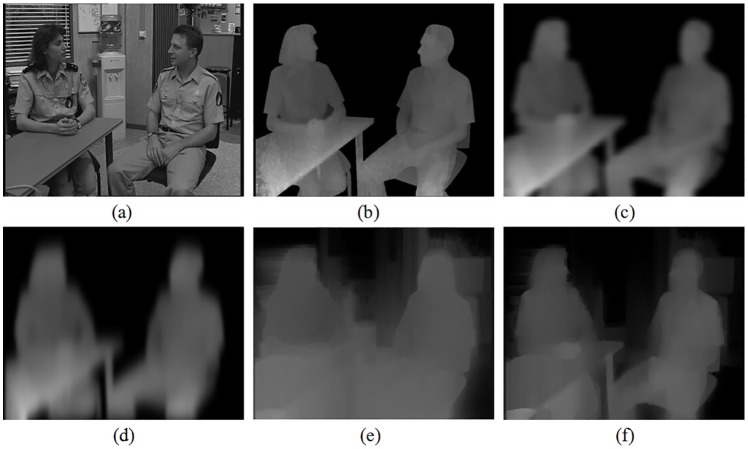
Comparison of smoothed depth maps using different *all blur* solutions. A: original texture image, B: original depth map, C: symmetric filter, D: asymmetric filter, E: proposed filter with constraint *ct*_1_(*u*), F: proposed filter with constraints *ct*_1_(*u*) and *ct*_2_(*u*).

Without loss of generality, for showing implementation details of our method, the tested set *Interview* is mainly analyzed in the following. This test set is with resolutions of 720 × 576. As discussed in Eq (6), our filter incorporates three different constraints in the transformed domain. To exploit the processing details of the proposed method, we only take the previous two constraints into considerations firstly. And at this time, the filter temporarily turns into an *all blur* filter. The experimental results are presented in Fig 4. Here we compare it with two other different *all blur* solutions.

Fig 4B is the related original depth map of Fig 4A. It can be observed that although the depth map is of highly consistent with the texture image in this test set, sharp depth discontinuities, which may lead to hole occurrences as displayed in the following experiments, exist at important object boundaries. As shown in Fig 4C and 4D, asymmetric Gaussian filter is more advanced than the traditional symmetric Gaussian filter in the 2D to 3D conversion thanks to the stronger smoothing in vertical direction for alleviating the geometric distortion caused by the vertical edge lines. Even so, compared to our *data-driven* structure-aided joint filter, the smooth strengths of these Gaussian filters are global unique. In fact, the sharpness of depth edge discontinuities in a depth map can not be always consistent. Based on that, severe distortions may still not be completely avoided with these global setting filters. As shown in Fig 4E, when our proposed filter only considers the first constraint *ct*_1_(*u*) in the transformed domain, we can see that the smooth strength is adjusted adaptively at different parts according to the structure information around. Noticing in the background of the woman’s head, the smooth strength is mainly appeared horizontally, which is consistent with the structure of the window shades behind. While in the background of the man’s head, the smooth strength is mainly appeared vertically. This is consistent with the structure features of the door behind. Experiments show that the smoothed depth map can simultaneously avoid geometric distortions by alleviating sharp depth discontinuities in two parts of different directions respectively in a united framework. To achieve the similar effect with these global Gaussian filters, an additional complex filter selection step must be designed such as previous works [27]. In Fig 4F, when our proposed filter considers both the first constraint *ct*_1_(*u*) and the second constraint *ct*_2_(*u*) in the transformed domain, depth map smooth is further optimized with the help of the visual saliency information. Comparing Fig 4E and 4F, we notice that, on the one side, the over-smooth at the bottom of the processed depth map is obviously reduced. On the other side, the filtering effects around the human body, where the predicted holes are mainly distributed, are still preserved. In a word, the perfect optimization to the smoothed depth map benefits from the second term *ct*_2_(*u*) in our domain transform framework.

**Fig 5:**
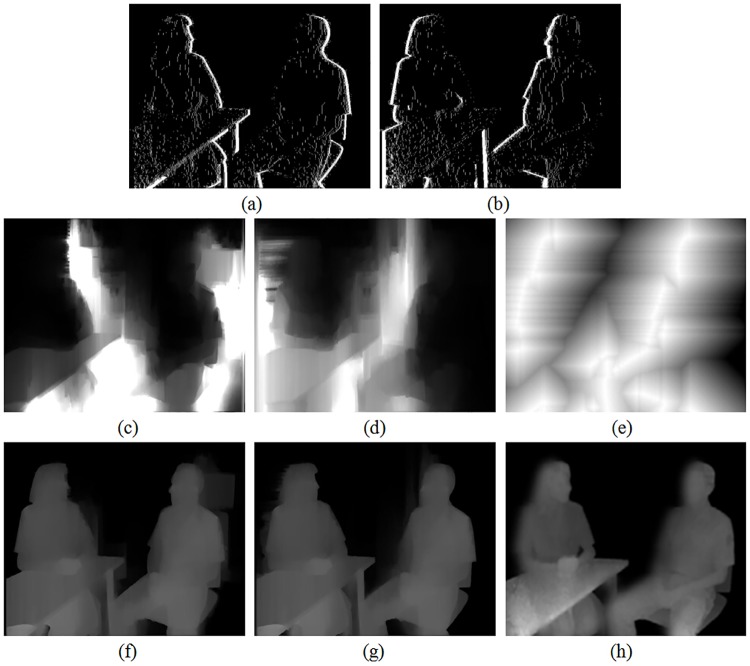
Experimental results of different depth map fusion solutions. A: detected hole regions in a right view, B: detected hole regions in a left view, C and F: influence map and processed depth map using our proposed method for a right view, D and G: influence map and processed depth map using our proposed method for a left view, E and H: influence map and processed depth map with method [20] for a left view.

When constraint *ct*_3_(*u*) is taken into consideration, the proposed domain transform filter further limits the filtered regions. In this part, our solution is compared with other similar method [20]. As shown in Fig 5A, if the original image of test set is set as a left view, then for a right view, the areas of newly exposed holes are located along the right side of foreground objects. Vice versa, for a left view, holes are located along the left side as presented in Fig 5B. Fig 5C and 5D are the influence maps generated with the process as we have discussed in Eq (14). And the corresponding processed depth maps are presented in Fig 5F and 5G.

From these results, several observations can be made. First, the filtered regions determined by the influence maps of our method are more adaptive than the edge-oriented method. From Fig 5E, we can see that the filtered regions are symmetric along some edges in the depth map horizontally. While in our method, they distribute adaptively according to the corresponding structure information around. For example, in Fig 5D, the influence map spreads vertically around the background of the man’s head, so that the distortions for the vertical lines in this area can be further reduced. Second, our filter can realize local smoothness in a united framework. As shown in Fig 5F and 5G, the effect of our method is similar to the edge-oriented method in Fig 5H. The filtered regions are mainly along the edge sides where disocclusion occurs. So compared to the results with *all blur* solutions in Fig 4, in the non-hole regions unnecessary smoothing process of depth maps is alleviated and the original resolution of depth maps is preserved. Therefore, good quality 3D images can be generated.

**Fig 6:**
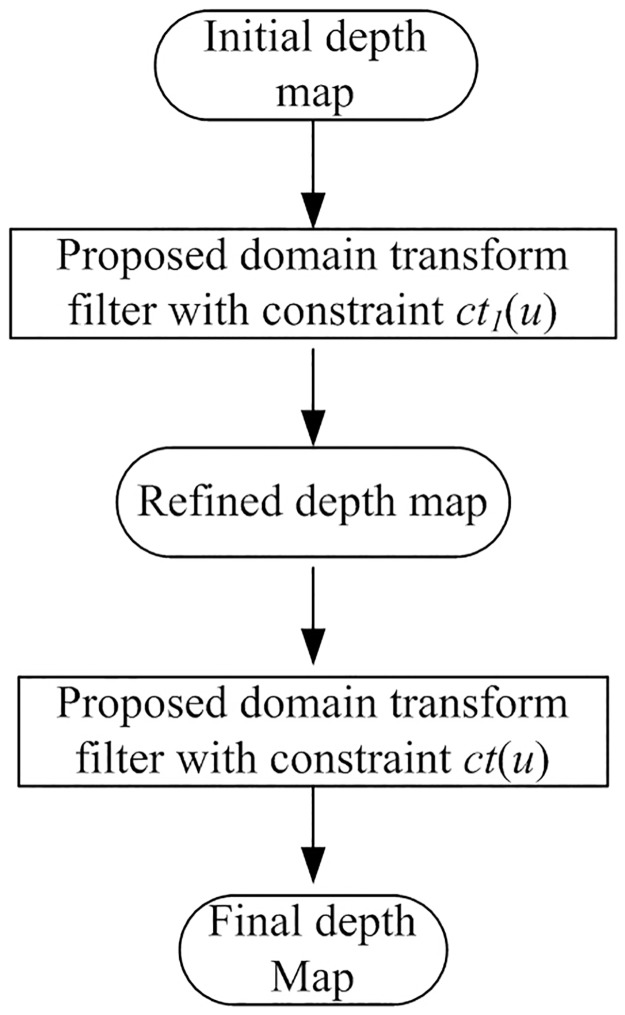
Processing flow for smoothing severely inaccurate depth maps with the proposed filter.

**Fig 7:**
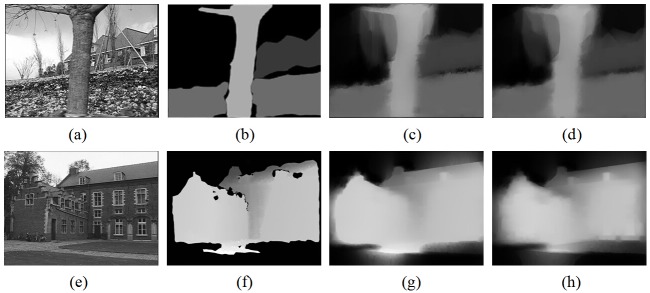
Experimental results of optimized depth maps with our proposed filter. A and E: original images, B: initial depth map of [25] (*flower* set) / F: initial depth map of [26] (*castle* set), C and G: refined depth maps, D and H: final optimized depth maps.

As discussed in last section, our filter can realize depth correction when preprocessing depth maps with inaccurate depth edges. To better evaluate its performance, in this part, we do experiments without the test sets *Interview* of accurate natural depth maps. Instead, two other test sets with severely inaccurate depth maps generated by automatic 2D to 3D conversion methods are adopted to test the robustness and reliability of our proposed framework. For the first test sets *Flower*, initial depth maps are generated by the cues from *Depth From Motion* [25], and for the second test sets *Castle*, initial depth maps are generated through simple *Delaney Triangulation* by the cues from *Structure From Motion* [26]. As shown in Fig 7B and 7F, due to severely inaccurate depth edges, traditional filters can not smooth these initial depth maps directly without any extra depth refinement step. But we can use our proposed domain transform framework to realize depth correction and smoothing simultaneously. The processing flow is displayed in Fig 6. Because in this situation, we can not predict available hole occurrences by Eq (11) based on inaccurate initial depth maps directly, the proposed domain transform filter firstly smoothes the initial depth map only with the constraint *ct*_1_(*u*). After that, we get the refined depth maps as shown in Fig 7C and 7G. From the experimental results, we can see that the quality of the refined depth maps have been improved greatly, especially for depth edges which have been of great consistence with the edge lines in related texture images. With the refined depth maps, now the disocclusion holes can be predicted. Finally, using the proposed all-constraints domain transform filter in Eq (6), we get the secondly optimized depth maps with the refined depth maps as the source images to be processed. The final optimized depth maps are given in Fig 7D and 7H. From these experimental results, we can see that the improvements between the refined depth maps and the final optimized depth maps are beneficial, but limited. The reasons behind this can be analyzed more clearly: after the first smoothing with our filter, sharp depth edges are reduced greatly, then the alterations of depth structures in the refined depth maps can further lead to the alleviation of hole occurrences in the hole prediction step of the second smoothing. For these reasons, the smooth strengths in the final depth maps are limited adaptively by our filter.

Actually, when processing severely inaccurate depth maps with our proposed filter, the refined depth map can always be regarded as the final processed depth map. In this way, the processing flow in Fig 6 can be simplified.

### Virtual view evaluation

**Fig 8:**
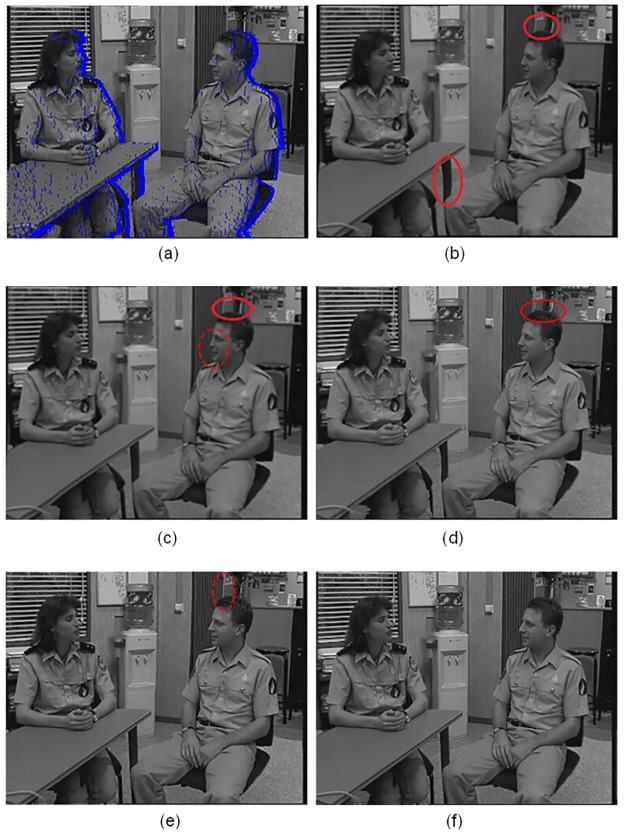
Virtual view images of *Interview* (720 × 576 pixels). A: no preprocessing, B: symmetric filter, C: asymmetric filter, D: distance dependent filter, E: proposed filter 1, F: proposed filter 2.

**Fig 9:**
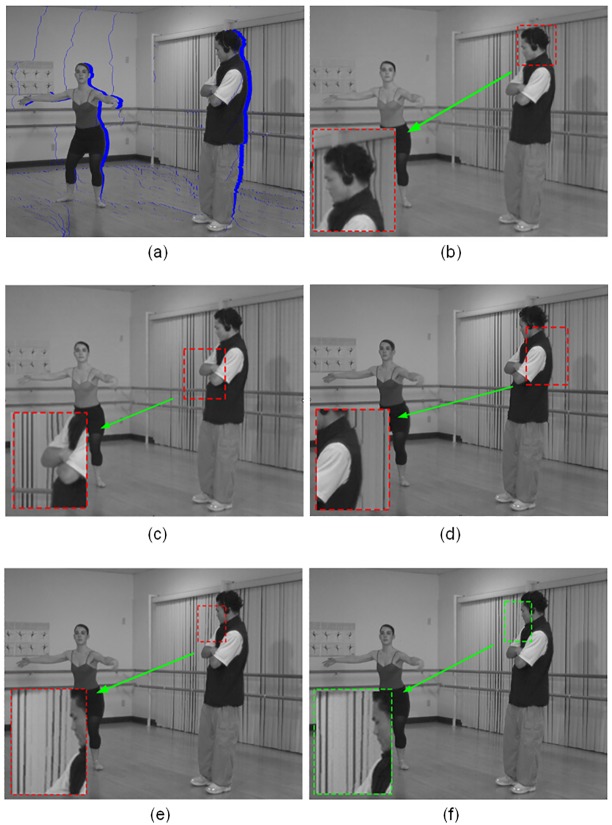
Virtual view images of *Ballet* (1024 × 768 pixels). A: no preprocessing, B: symmetric filter, C: asymmetric filter, D: distance dependent filter, E: proposed filter 1, F: proposed filter 2.

In this part, besides of some aforementioned intermediate results, the final output synthesized by the proposed methods and some other view synthesis methods are firstly presented. As discussed in the above sub-section, for test sets *Flower* and *Castle*, our method can preprocess the inaccurate depth maps directly, while other traditional filters must employ extra refinement steps before formal processing, which has beyond the discussions in this paper. For this reason, in this part, we only compared the visual quality of the synthesized virtual view images with different methods for test sets *Interview* and *Ballet*. Series of virtual view results are provided in Figs 8 and 9. The blue regions in Figs 8A and 9A represent the disocclusion areas. *Proposed filter 1* means the proposed filter without constraint *ct*_3_(*u*), and *proposed filter 2* means the proposed all-constraint filter.

From these results, several observations can be made. First, generally speaking traditional *all blur* methods are more inclined to cause geometric distortions. As shown in the red circles of Fig 8B and 8C and red rectangles of Fig 9B and 9C. Although asymmetric filter can reduce the phenomenon, additional side-effect such as over-smooth in the red dot circle of Fig 8C is introduced. Our *proposed filter 1* can also be regarded as an *all blur* method. Due to using adaptive structure-aided setting instead of the global setting, the geometric distortions in Fig 8E are not obvious, and even better in Fig 9E. Second, partial smooth methods, such as distance dependent filter and our *proposed filter 2*, have advantages on preserving the original image quality in the non-hole regions. As shown in Figs 8D, 8F, 9D and 9F, thanks to the limitations of smoothing regions, the details in the backgrounds are more clear than the results generated by *all blur* methods in Figs 8B, 8C and 9B and 9C. Third, methods with our *proposed filter 2* give better quality of the virtual views compared to other algorithms, even in the marked areas of Figs 8D and 9D, where the distance dependent filter can not process well. These advantages can be more clearly displayed in the following PSNR and SSIM comparison. In a word, the proposed methods in this paper are suitable to synthesize high quality stereoscopic virtual view images for the 2D to 3D conversion.

The synthesized images can be evaluated by PSNR comparison [20]. The PSNR value for each method is obtained by calculating the average MSE between the preprocessed depth map and the original depth map with the mask where the disocclusion areas are excluded. Similarly, the SSIM metric [28] is also used in this paper, and in the output result, we only measure the areas of depth maps with the mask where the disocclusion areas are excluded. We can observe on Table 2 the important quality improvement obtained with the proposed method. Table 3 shows the computation times of different methods. From these tables several observations can be made. First, generally speaking, it turns out that our method is both excellent at time saving and virtual view quality. Second, our methods take the least computation times against other methods, the high efficiency mainly benefits from the proposed domain transform framework. Third, partial smooth methods always get higher PSNR and SSIM scores than *all blur* methods. In should be noticed that although distance dependent filter even get higher PSNR and SSIM scores in test set *Interview* than our *proposed filter 2*, and in test set *Ballet* than our *proposed filter 1*, these indicators only supply certain references to evaluate virtual views. As shown in Figs 8D, 8F, 9D and 9E, in fact, our proposed methods realize less distortions and better optimizations than their counterparts by structure-aided smooth, which are more important for virtual view quality, and cannot be demonstrated directly by depth map PSNR or SSIM comparisons. The following subjective evaluation in Table 4 verifies the observation.

**Table 2:** PSNR and SSIM comparison.

	Test set	Symmetric filter	Asymmetric filter	Distance dependent filter	Proposed filter 1	Proposed filter 2
PSNR(dB)	Interview	28.62	29.94	31.36	30.33	31.14
	Ballet	28.28	29.53	31.13	30.96	31.19
SSIM	Interview	0.8865	0.8941	0.9245	0.9021	0.9187
	Ballet	0.8732	0.8876	0.9216	0.9164	0.9241

**Table 3:** Computation time (s) comparison.

Test set	Symmetric filter	Asymmetric filter	Distance dependent filter	Proposed filter 1	Proposed filter 2
Interview	0.83	1.19	0.31	0.16	0.23
Ballet	0.98	1.27	0.38	0.18	0.26

**Table 4:** Results of subjective quality evaluation.

	Test set	Symmetric filter	Asymmetric filter	Distance dependent filter	Proposed filter 1	Proposed filter 2
Test1	Interview	3.8	4.1	4.3	4.4	**4.5**
	Ballet	3.9	4.2	4.3	4.3	**4.6**
Test2	Interview	3.7	3.9	4.1	4.2	**4.3**
	Ballet	3.8	4.1	4.1	**4.3**	**4.3**

### Results of subjective quality evaluation

**Fig 10:**
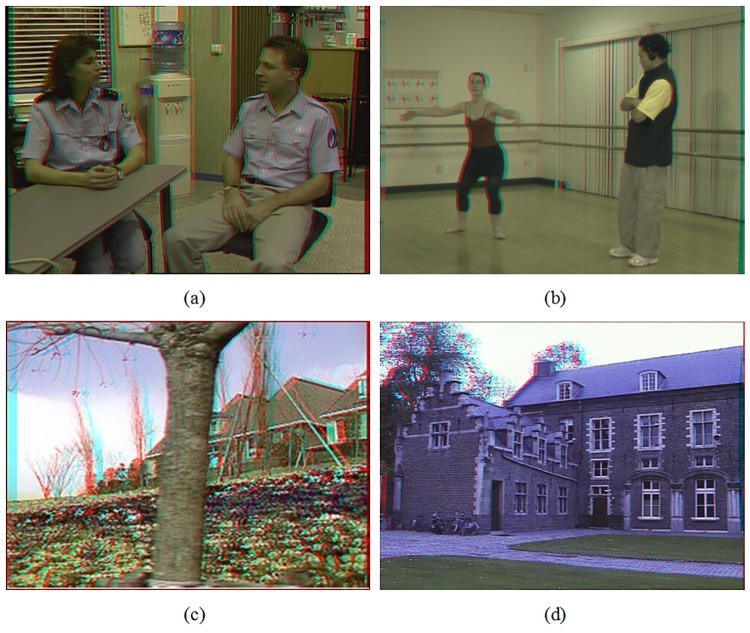
Synthesized anaglyph images of test sets. A: *Interview*, B: *Ballet*, C: *Flower*, D: *Castle*.

Subjective viewing tests are also performed with these test sets by 15 individuals with normal or correct-to-normal visual acuity and stereo acuity. We made two tests to evaluate image quality of the newly generated virtual views and stereoscopic feeling of the synthesized 3D anaglyph images separately. Both of the scores were from 0 to 5, and a higher score illustrates higher image quality or stereoscopic feeling. In the first test, the participants watched the original images in each test set to have a high reference before formal evaluation. Also in the second test, before the formal evaluation, training is given to the participants using true 3D anaglyph images to help them gain a better understanding of the stereoscopic feeling. In the tests, test images of each set are displayed in a random order. The average score was obtained and used as a measure of the subjective evaluation as shown in Table 4. The best results are high lighted with bold face type. From this table, we can see that the proposed methods present better performances in both image quality on the newly generated virtual views and stereoscopic feeling on the synthesized 3D anaglyph images. We also notice that the scores of Test 2 were lower than those of Test 1 in most cases. One reason is that hole filling is a challenging problem, and it is made more difficult with stereoscopic content as image features need to have consistent disparity across binocular views. The other reason, especially for the test images using the traditional all blur smoothing methods, is that although the visual quality of the new generated virtual views is improved, the depth perception of the scene at important (salient) object edges is still distorted due to additional disparity shifts when a filter smoothes the depth map. In this paper, our proposed methods have special constraints to reduce distortions for these situations. Fig 10 shows some examples of the synthesized 3D anaglyph images in the evaluation test sets.

## Conclusions

This paper presents a new depth map preprocessing method based on structure-aided domain transform smoothing for the DIBR system. The proposed scheme, which builds the framework in the transformed domain and combines different cues in a united form, can handle the problems of disocclusion occurrences, edge inconsistency and over-smoothness in the DIBR process simultaneously. Benefit from the high efficiency of domain transform framework, our methods can prevent holes before DIBR via taking multiple related constraints into considerations to exploit optimally and adaptively structure-aided smoothness of a typical depth map. Experimental results have illustrated the high efficiency of the proposed method. The method can be applied not only in 2D to 3D conversion, but also for any type of 3D synthesis applications. It should be pointed out that at present, the proposed methods in this paper synthesize a stereoscopic image only with an original texture image and its corresponding depth map. When using these methods in synthesis applications to videos, temporal correlation between different frames should be considered to further guarantee the video temporal stability. And a temporal filtering procedure for the input sequence is beneficial. To extend our methods for stereoscopic video synthesis, these improvements would be the focuses of our further research work.

## Supporting information

S1 Fig Relevant data underlying the findings described in the experimence of Fig 4. (PPT)

S2 Fig Relevant data underlying the findings described in the experimence of Fig 5. (PPT)

S3 Fig Relevant data underlying the findings described in the experimence of Fig 7. (PPT)

S4 Fig Relevant data underlying the findings described in the experimence of Fig 8. (PPT)

S5 Fig Relevant data underlying the findings described in the experimence of Fig 9. (PPT)

S6 Fig Relevant data underlying the findings described in the experimence of Fig 10. (PPT)
