# Supplementary figures and images for: An efficient depth map preprocessing method based on structure-aided domain transform smoothing for 3D view generation
Source: PLoS One. 2017 Apr 13;12(4):e0175910. doi: 10.1371/journal.pone.0175910 (PMC5391110; doi:10.1371/journal.pone.0175910)

## Slide 1
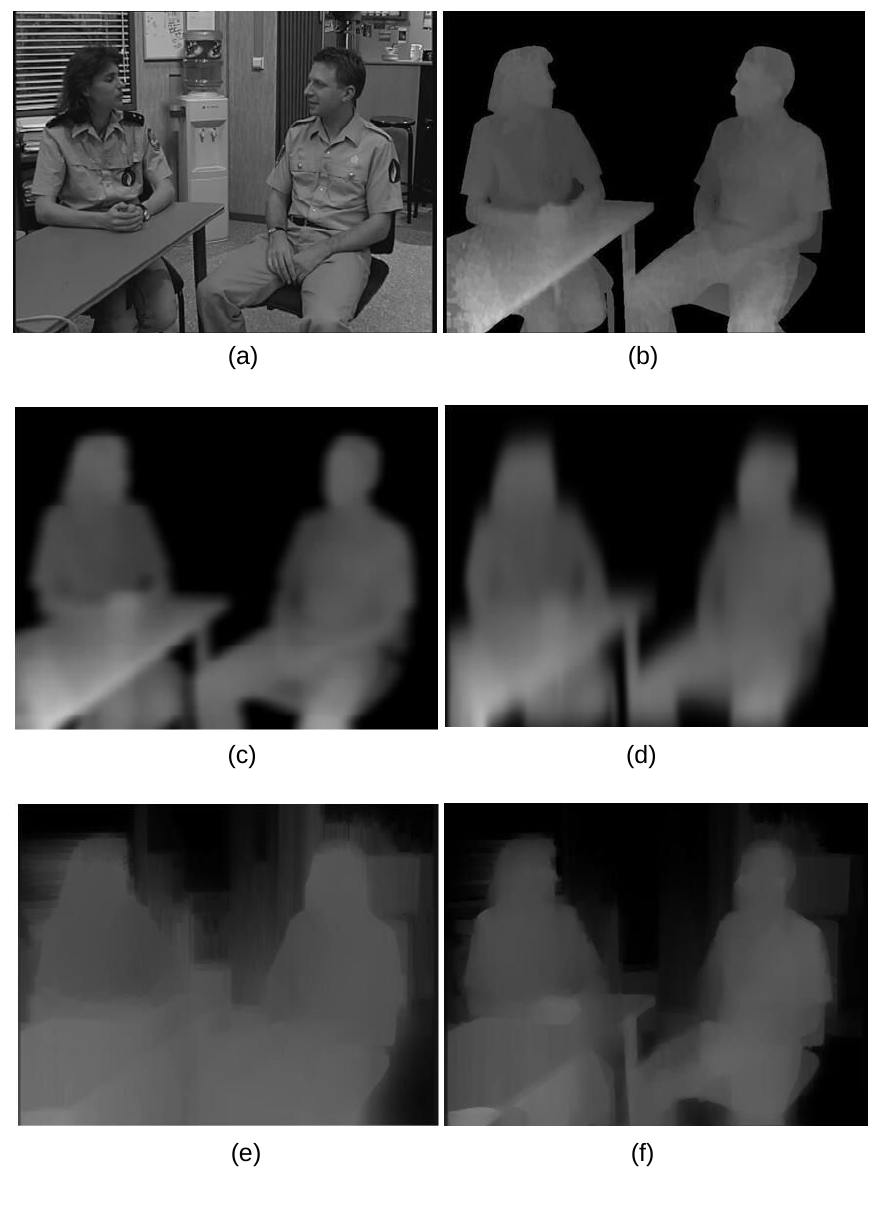

(a) (b)
(c) (d)
(e) (f)

Supplement: S1 Fig — (PPT) [file pone.0175910.s001.ppt]

## Slide 1
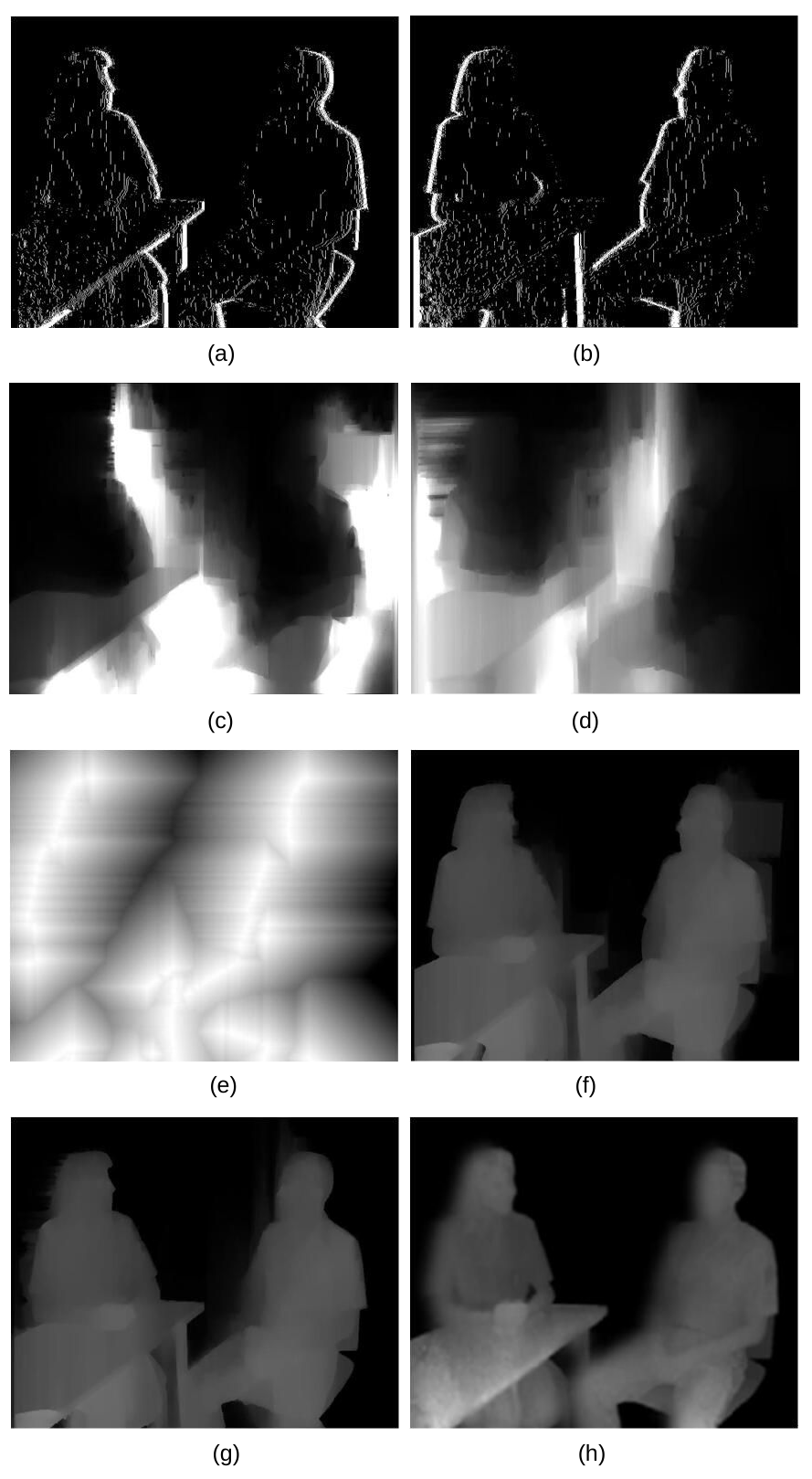

(a) (b)
(c) (d)
(e) (f)
(g) (h)

Supplement: S2 Fig — (PPT) [file pone.0175910.s002.ppt]

## Slide 1
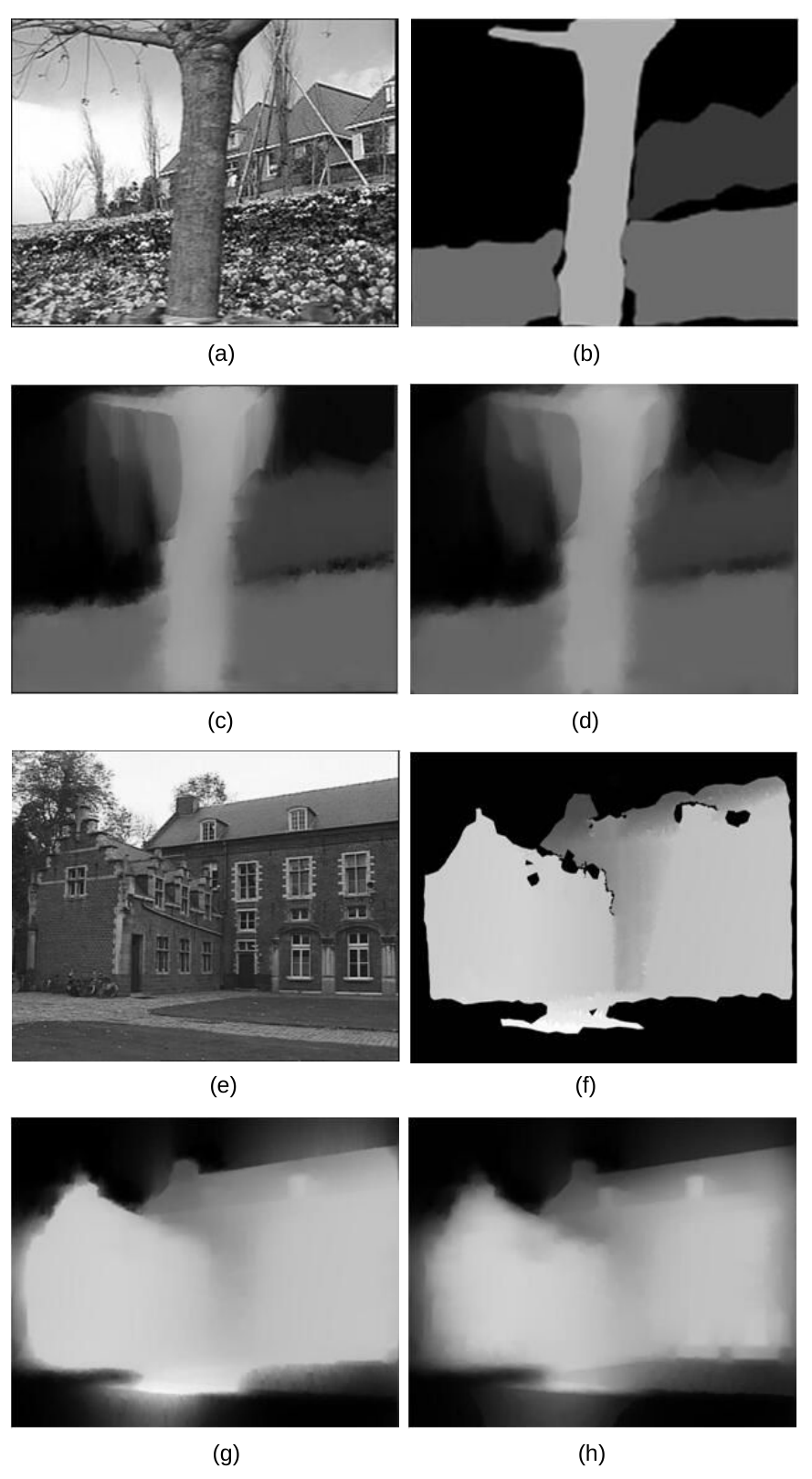

(a) (b)
(c) (d)
(e) (f)
(g) (h)

Supplement: S3 Fig — (PPT) [file pone.0175910.s003.ppt]

## Slide 1
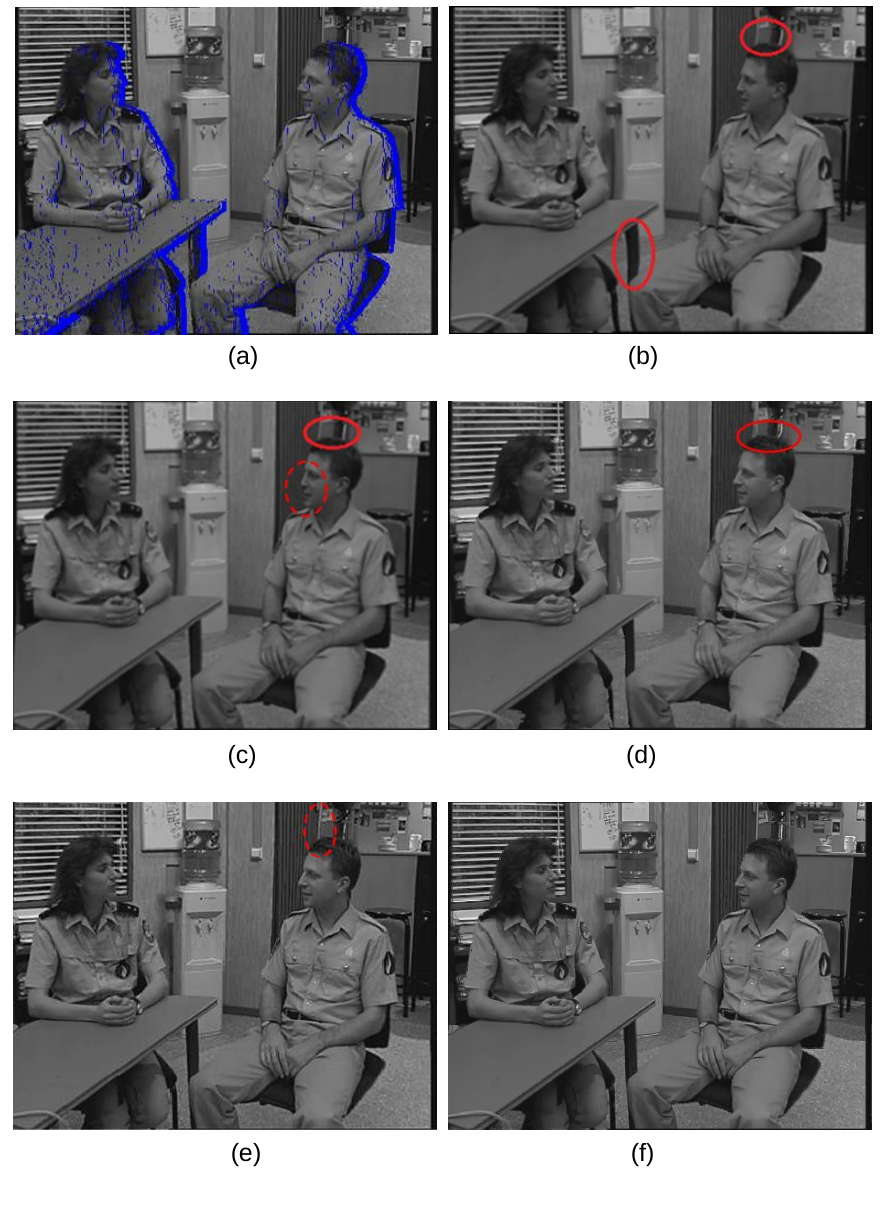

(a) (b)
(c) (d)
(e) (f)

Supplement: S4 Fig — (PPT) [file pone.0175910.s004.ppt]

## Slide 1
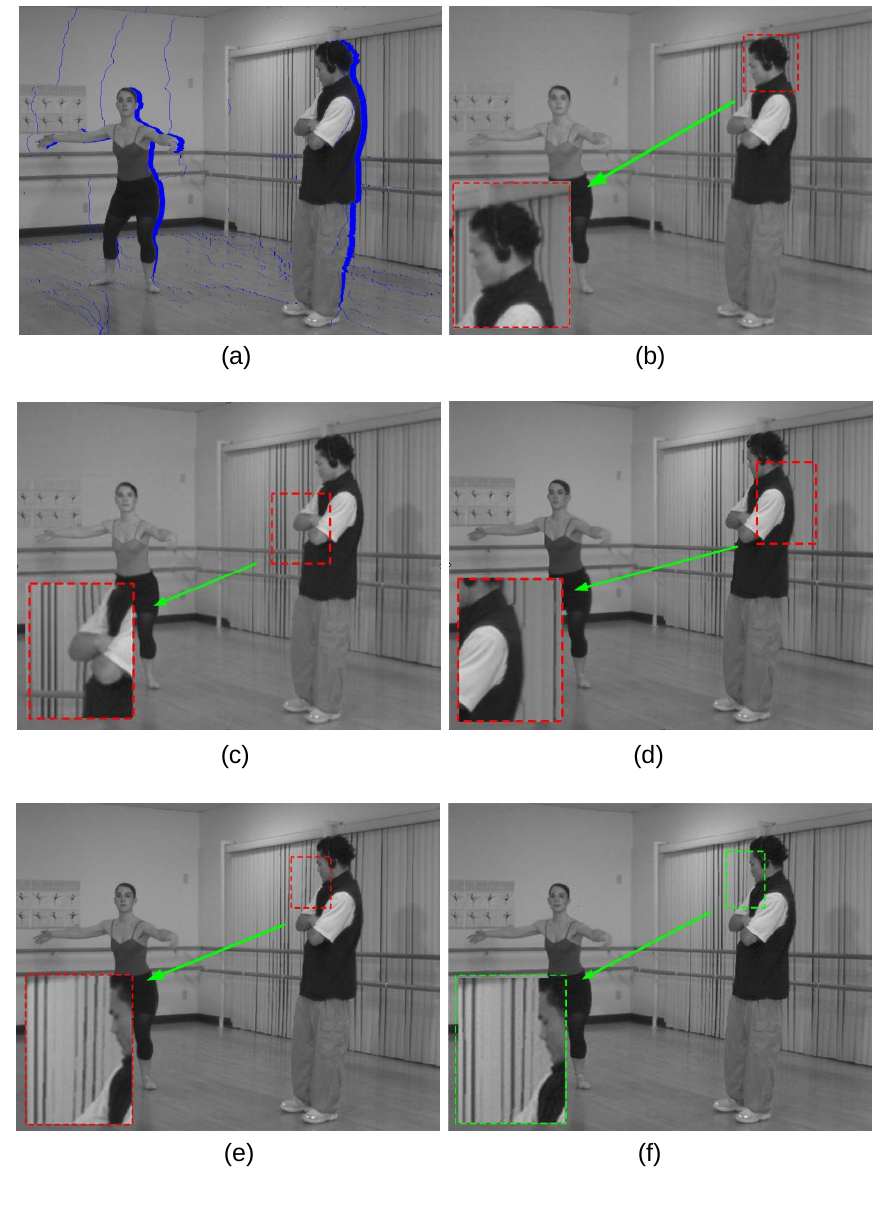

(a) (b)
(c) (d)
(e) (f)

Supplement: S5 Fig — (PPT) [file pone.0175910.s005.ppt]

## Slide 1
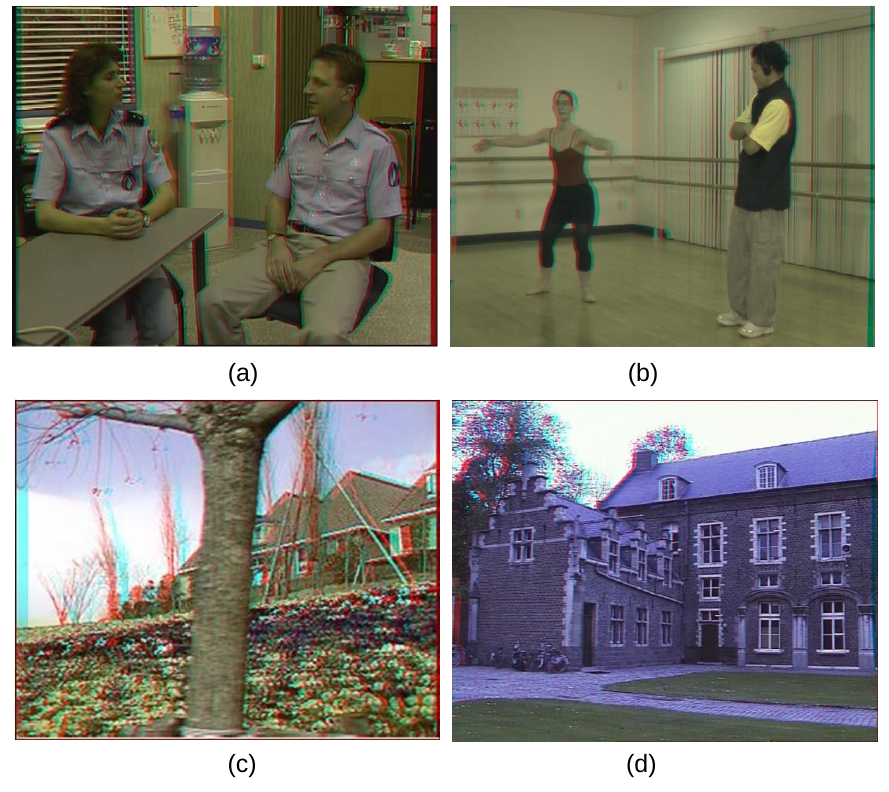

(a) (b)
(c) (d)

Supplement: S6 Fig — (PPT) [file pone.0175910.s006.ppt]
